# Common polymorphism in *H19 *associated with birthweight and cord blood IGF-II levels in humans

**DOI:** 10.1186/1471-2156-6-22

**Published:** 2005-05-10

**Authors:** Clive J Petry, Ken K Ong, Bryan J Barratt, Diane Wingate, Heather J Cordell, Susan M Ring, Marcus E Pembrey, The ALSPAC Study Team, Wolf Reik, John A Todd, David B Dunger

**Affiliations:** 1 Department of Paediatrics, University of Cambridge, Addenbrooke's Hospital Level 8, Box 116, Cambridge CB2 2QQ, UK; 2 Juvenile Diabetes Research Foundation/Wellcome Trust Diabetes and Inflammation Laboratory, Cambridge Institute for Medical Research, University of Cambridge, Addenbrooke's Hospital, Cambridge, UK; 3 Unit of Paediatric and Perinatal Epidemiology, University of Bristol, 24 Tyndall Avenue, Bristol, UK; 4 Clinical and Molecular Genetics Unit, Institute of Child Health, London, UK; 5 Laboratory of Developmental Genetics and Imprinting, Babraham Institute, Cambridge, UK

## Abstract

**Background:**

Common genetic variation at genes that are imprinted and exclusively maternally expressed could explain the apparent maternal-specific inheritance of low birthweight reported in large family pedigrees. We identified ten single nucleotide polymorphisms (SNPs) in *H19*, and we genotyped three of these SNPs in families from the contemporary ALSPAC UK birth cohort (1,696 children, 822 mothers and 661 fathers) in order to explore associations with size at birth and cord blood IGF-II levels.

**Results:**

Both offspring's and mother's *H19 *2992C>T SNP genotypes showed associations with offspring birthweight (P = 0.03 to P = 0.003) and mother's genotype was also associated with cord blood IGF-II levels (P = 0.0003 to P = 0.0001). The offspring genotype association with birthweight was independent of mother's genotype (P = 0.01 to P = 0.007). However, mother's untransmitted *H19 *2992T allele was also associated with larger birthweight (P = 0.04) and higher cord blood IGF-II levels (P = 0.002), suggesting a direct effect of mother's genotype on placental IGF-II expression and fetal growth. The association between mother's untransmitted allele and cord blood IGF-II levels was more apparent in offspring of first pregnancies than subsequent pregnancies (P-interaction = 0.03). Study of the independent Cambridge birth cohort with available DNA in mothers (N = 646) provided additional support for mother's *H19 *2992 genotype associations with birthweight (P = 0.04) and with mother's glucose levels (P = 0.01) in first pregnancies.

**Conclusion:**

The common *H19 *2992T allele, in the mother or offspring or both, may confer reduced fetal growth restraint, as indicated by associations with larger offspring birth size, higher cord blood IGF-II levels, and lower compensatory early postnatal catch-up weight gain, that are more evident among mother's smaller first-born infants.

## Background

The maternal-uterine environment has a major influence on fetal growth and size at birth [1]. Early cross-breeding experiments in animals demonstrated that fetal growth could be restrained or enhanced from its genetic potential, according to the size of the mother [2,3]. In a normal human birth cohort study, we demonstrated that over 50% of all offspring may have experienced clinically significant restraint or enhancement of growth *in utero*, followed by compensatory catch-up or catch-down growth respectively during the first 2 years of postnatal life [4].

Birth order is an important common determinant of maternal-uterine restraint, as first-born infants are more likely to be restrained *in utero *and show compensatory postnatal catch-up growth, compared to subsequent-born infants [5]. Heritable or genetic factors may also contribute to degree of maternal restraint of fetal growth; from studies of birthweights in extended human families it has been suggested that low birthweight may be maternally transmitted [6]. We previously reported association between thinness at birth and the common 16189 variant in mitochondrial DNA, which is maternally transmitted [7]. Another possible mechanism to explain maternal inheritance of fetal growth restraint is common variation in genes that are imprinted and exclusively maternally expressed.

Many of the genes known to be imprinted and paternally or maternally expressed influence fetal growth [8]. An imprinted region on chromosome 11p15.5 in humans regulates the major fetal growth factor "insulin-like growth factor-II" (IGF-II). The *IGF2 *gene is imprinted and paternally expressed in both the mouse and human fetus [9,10]. The mechanisms of imprinting and expression of *Igf2 *have been thoroughly studied in the mouse. The gene is regulated by DNA elements close to *Igf2*, such as silencers [11] and activators [12], and particularly by enhancers located distal to the neighbouring maternally expressed gene *H19 *[13,14]. Access to these enhancers is restricted by an epigenetically controlled insulator upstream of *H19 *[15-18]. Finally, the maternally expressed *H19 *gene itself does not code for a protein, but the RNA has growth suppressing functions [19], potentially through inhibiting translation of IGF2 RNA [20].

We therefore aimed to demonstrate whether common genetic variation in human *H19 *may be associated with fetal growth restraint in a large representative birth cohort. We identified a common *H19 *2992 C>T SNP that was associated with offspring birth size, cord blood IGF-II levels, and infancy catch-up weight gain. These associations were more evident among first-born infants, and we propose that the common *H19 *2992T allele may confer reduced fetal growth restraint.

## Results

Of the ten SNPs that we identified in the *H19 *region, we selected three SNPs (2992 C/T; 1737 A/G; and 3238 A/G) that marked different common *H19 *haplotypes for genotyping in ALSPAC offspring DNA samples for genetic association studies.

### Associations with offspring *H19 *genotypes

Offspring *H19 *2992 SNP genotypes showed significant associations with size at birth (Table 1). However, neither *H19 *1737 nor 3231 genotypes were associated with birth size (data not shown).

*H19 *2992, 1737 and 3231 genotypes were in significant linkage disequilibrium, and haplotype analysis revealed only 7 genotype combinations with frequency >1%. Only those two haplotypes that included a 2992T allele had birthweights that were above the average [see Additional file 1].

**Table 1:** Birth size and cord IGF-II levels by offspring *H19 *2992 genotype (ALSPAC cohort)

	**CC**	**CT**	**TT**	P_A_	P_D_
	*(n = 1075)*	*(n = 518)*	*(n = 54)*		
Weight (gm)	3468 ± 488	3522 ± 486	3524 ± 587	0.03	0.02
Length (cm)	50.7 ± 2.0	50.9 ± 2.0	50.9 ± 2.1	0.08	0.06
Head circ. (cm)	34.9 ± 1.2	34.9 ± 1.3	35.0 ± 1.6	0.3	0.4
	*(n = 226)*	*(n = 110)*	*(n = 13)*		
Cord IGF-II (ng/dl)	275 ± 78	277 ± 79	337 ± 91	0.1	0.3

Mean ± SD, adjusted for sex, gestation and parity.

P_A _= P value for additive genetic model (per T allele)

P_D _= P value for dominant genetic model (CC versus T/*)

### Associations with mother's *H19 *2992 genotype

**Figure 1:**
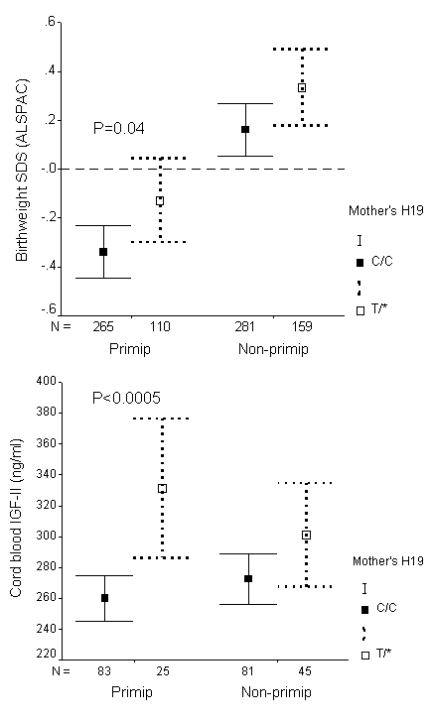
Birthweight SD score (A) and cord blood IGF-II levels at birth (B) in the ALSPAC cohort, by mother's *H19 *2992 genotype, and stratified by birth order ("Primip" = mother's first child; "Non-primip" = second or subsequent child). Mean ± 95% CI. First-born infants had lower birthweights than infants of subsequent pregnancies (Ref. 5). Associations with mother's genotype (CC vs. T* [CT or TT]) were only seen in first pregnancies.

The *H19 *2992 SNP was subsequently genotyped in DNA samples collected from mothers and fathers, for which the family relationships had been validated by genotyping. *H19 *2992 genotype in mothers, but not in fathers, showed associations with size at birth, and also with cord blood IGF-II levels (Table 2). Mother's *H19 *2992 genotype associations were independent of mother's pre-pregnancy weight and height (additive models: birthweight: P = 0.001; cord IGF-II levels: P = 0.0005). The association between mother's *H19 *2992 genotype and IGF-II levels appeared to vary with mother's parity (birth order), being evident only in first pregnancies (Figure 1), although formal test for interaction did not reach significance (P = 0.06). When both mother's and offspring's genotypes were included in a multivariate analysis, birthweight was significantly associated with both mother's *H19 *2992 genotype (P < 0.05) and also offspring's *H19 *2992 genotype (P = 0.01), suggesting that there were separate effects of mother's and offspring's genotype on birthweight.

**Table 2:** Birth size by mothers or fathers *H19 *2992 genotype (ALSPAC cohort)

*Mothers genotype*	**CC**	**CT**	**TT**	P_A_	P_D_
	*(n = 550)*	*(n = 242)*	*(n = 30)*		
Weight (gm)	3457 ± 474	3543 ± 508	3595 ± 485	0.003	0.003
Length (cm)	50.7 ± 2.0	51.0 ± 1.9	51.1 ± 1.8	0.04	0.04
Head circ. (cm)	34.8 ± 1.2	35.1 ± 1.3	34.9 ± 1.4	0.07	0.03
	*(n = 164)*	*(n = 61)*	*(n = 9)*		
Cord IGF-II (ng/dl)	266 ± 72	312 ± 99	312 ± 92	0.0003	0.0001
					
*Fathers genotype*	**CC**	**CT**	**TT**	P_A_	P_D_
	*(n = 442)*	*(n = 201)*	*(n = 18)*		
Weight (gm)	3492 ± 491	3474 ± 472	3537 ± 519	0.9	0.7
Length (cm)	50.8 ± 1.9	50.8 ± 1.9	51.1 ± 1.7	0.8	0.9
Head circ. (cm)	34.9 ± 1.2	34.9 ± 1.2	35.0 ± 1.2	0.9	0.7
	*(n = 76)*	*(n = 37)*	*(n = 3)*		
Cord IGF-II (ng/dl)	270 ± 75	290 ± 93	245 ± 96	0.5	1.0

Mean ± SD, adjusted for sex, gestation and parity.

P_A _= P value for additive genetic model (per T allele)

P_D _= P value for dominant genetic model (CC versus T/*)

### Mother's untransmitted *H19 *2992 allele effects

Effects of mother's genotype, independent of transmission to the offspring, were examined by looking at association with mother's *H19 *2992 CC versus CT genotype within CC genotype offspring. Mother's untransmitted T versus C allele was associated with larger birthweight (P = 0.04) and higher cord blood IGF-II levels (P = 0.002; Table 3). As suggested by the overall associations with mother's genotype (in Figure 1), the effect of mother's untransmitted allele on IGF-II levels was clearer in first pregnancies (P = 0.0002) than in subsequent pregnancies (P = 0.36; P-interaction between mother's untransmitted allele and birth order = 0.03).

**Table 3:** Birthweight and IGF-II levels by maternal *H19 *2992 genotype, within CC genotype offspring (i.e. effect of mother's untransmitted allele), (ALSPAC cohort)

	**Mother's Genotype**		ANOVA
	**CC**	**CT**	
Birthweight (gm)	3431 ± 474	3533 ± 505	P = 0.04
	(n = 407)	(n = 96)	
Cord IGF-II (ng/dl)	264 ± 72	320 ± 108	P = 0.002
	(n = 124)	(n = 22)	

Mean ± SD, adjusted for sex, gestation and parity.

Those results indicate that the *H19 *2992 SNP associations may depend on mother's genotype. However, offspring *H19 *2992 SNP associations were also seen independent of mother's genotype; among CC genotype mothers, offspring CC vs. CT genotype was also associated with birthweight (P = 0.007, Table 4).

**Table 4:** Birthweight and IGF-II levels by offspring *H19 *2992 genotype, within CC genotype mother's (i.e. effect of fetal genotype), (ALSPAC cohort)

	**Offspring Genotype**		ANOVA
	**CC**	**CT**	
Birthweight (gm)	3425 ± 490	3535 ± 495	P = 0.007
	(n = 408)	(n = 126)	
Cord IGF-II (ng/dl)	262 ± 80	266 ± 98	P = 0.8
	(n = 125)	(n = 36)	

Mean ± SD, adjusted for sex, gestation and parity.

### *H19 *2992 allele transmission and parent-of-origin effects

Among the validated parent-offspring trios, comparison of allele transmission from heterozygous parents to their offspring showed that T versus C allele transmission was not associated with birthweight (P = 0.22, number of observations = 204), nor with cord blood IGF-II levels (P = 0.52, number of observations = 38). Nor was there any difference between paternal and maternal T allele transmission (parent-of-origin effects) on birthweight (P = 0.92, number of observations = 87), or on IGF-II levels (P = 0.81, number of observations = 15). Data on mother's parents' genotypes were unavailable.

### Genotype associations with postnatal weight gain

**Figure 2:**
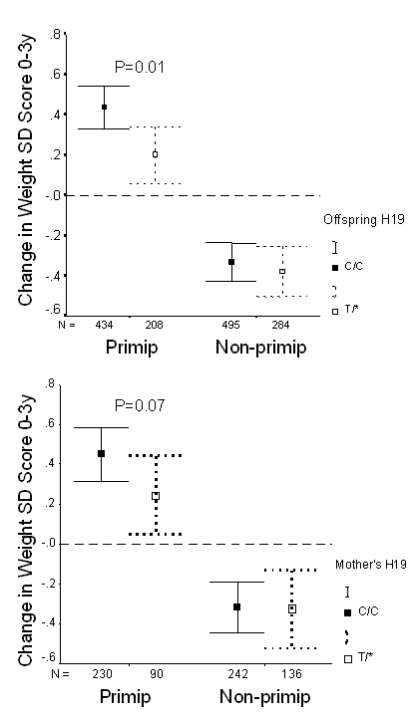
Postnatal weight gain (change in weight SD score 0–3 years) in the ALSPAC cohort, by offspring's (A) or mother's (B) *H19 *2992 genotype, and stratified by birth order ("Primip" = mother's first child; "Non-primip" = second or subsequent child). Mean ± 95% CI. The overall association between weight gain and offspring genotype (CC vs. T* [CT or TT], P = 0.01; Table 5) was only apparent in first-born offspring.

1,449 ALSPAC children were followed-up to age 3 years. Neither offspring nor parental *H19 *genotypes showed association with offspring body size at age 3 years (not shown). The apparent loss of T allele-related size advantage between birth and age 3 years was explained by slower rates of weight gain during this period (Table 5). As with birthweight and IGF-II levels, the *H19 *2992 genotype associations with postnatal weight gain were more apparent in offspring of first pregnancies (Figure 2).

**Table 5:** Size at 3 years, and growth from birth, by offspring *H19 *2992 genotype, (ALSPAC cohort)

*Offspring genotype*	**CC**	**CT**	**TT**	P_A_	P_D_
	(n = 946)	(n = 462)	(n = 41)		
Weight at 3 y (kg)	15.0 ± 1.7	14.9 ± 1.7	14.8 ± 1.7	0.4	0.4
Height at 3 y (cm)	95.6 ± 3.6	95.3 ± 3.5	95.2 ± 3.7	0.2	0.2
Change in Weight SDS 0–3 y	0.01 ± 1.17	-0.13 ± 1.07	-0.10 ± 1.29	0.01	0.02
Change in Height SDS 0–3 y	0.06 ± 1.12	-0.10 ± 1.02	-0.17 ± 1.26	0.01	0.01

Mean ± SD, adjusted for sex and age

P_A _= P value for additive genetic model (per T allele)

P_D _= P value for dominant genetic model (CC versus T/*)

### Replication of mother's *H19 *2992 genotype associations in the Cambridge cohort

**Figure 3:**
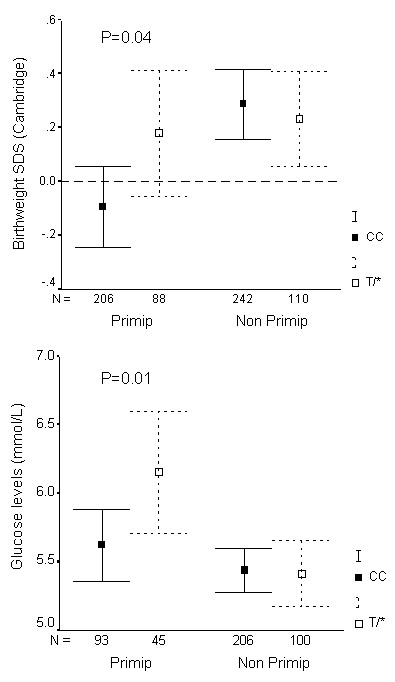
Birthweight SD score (A) and mother's glucose levels one hour post-oral glucose load at 27 to 32 weeks gestation (B) in the Cambridge cohort, by mother's *H19 *2992 genotype, and stratified by birth order ("Primip" = mother's first child; "Non-primip" = second or subsequent child). Mean ± 95% CI. Associations with mother's genotype (CC vs. T* [CT or TT]) were only seen in first pregnancies.

In the independent Cambridge birth cohort (n = 646 births analysed), we obtained additional support for the association between mother's *H19 *2992T allele and larger birthweight in offspring of first pregnancies (P = 0.04, Figure 3A). Mother's glucose levels one hour post oral glucose at 27 to 32 weeks gestation (mean ± SD: 5.5 ± 1.2 mmol/l) were positively related to offspring birthweight (r = 0.14, P = 0.01), and mother's *H19 *2992 T/* genotype was also associated with higher mother's glucose levels during first pregnancies (P = 0.01, Figure 3B).

## Discussion

In two separate contemporary birth cohorts we have identified associations between a common *H19 *2992 genotype and birthweight. This SNP is located in *H19 *exon 5 [see Additional file 2], however its effect on mRNA structure or function is unknown and *H19 *has no protein product. An insulator upstream of *H19 *[15-18] and enhancers downstream [13,14] regulate imprinting of *IGF2*, which encodes a major fetal growth factor [21]. In addition *H19 *RNA may suppress *IGF2 *expression in trans [20] and has tumour suppressor functions in cell transfection studies [19]. Consistent with the birthweight association, *H19 *2992 genotype was also associated with cord blood IGF-II protein levels at birth.

Birthweight associations were found with both mother's and offspring's *H19 *2992 genotypes, and it is not clear whether our findings represent direct effects of mother's genotype, offspring genotype, or both. As in other population studies, the low number of available DNA samples and genotypes from fathers contributed to relatively lower statistical power to formally detect effects of *H19 *2992 allele transmission. However, we did observe significant birthweight and cord blood IGF-II associations with mother's untransmitted allele, suggesting direct effects of mother's genotype. While in mice *H19 *and *IGF2 *are not expressed postnatally [22], in humans *IGF2 *expression continues into adulthood [23]. In most adult tissues *IGF2 *expression remains monoallelic as in the fetus, however biallelic expression is observed in adult human liver [23]. It is possible that mother's *H19 *2992 genotype may regulate *IGF2 *expression in maternally-derived placental tissues. Alternatively, *H19 *regulation of maternal *IGF2 *expression could influence mother's glucose metabolism [24] and thereby influence placental glucose transfer and fetal growth [25]. Offspring *H19 *2992 genotype was associated with birthweight, but not with IGF-II levels. It is possible that this discrepancy could relate to reduced power due to the smaller number of IGF-II samples available, or alternatively it could be possibly explained by a non-IGF-II mediated effect of *H19 *on birthweight. The association between birthweight and IGF-II levels in cord blood at birth is weak, and it is possible that the effects of IGF-II on fetal growth may be specific to certain tissues or developmental stages in fetal life [26].

The *H19 *2992 genotype associations with birthweight and IGF-II were independent of mother's body size and were more apparent in mother's first pregnancies. First-born offspring are more growth-restrained *in utero *than subsequent children, as evident by smaller birth size and compensatory rapid postnatal weight gain within the first 2 to 3 years [5]. The mechanism of growth restraint seen in first pregnancies is unknown, but could reflect a maternally inherited genetic trait with subsequent relaxation of restraint in later pregnancies [27]. We propose that the common *H19 *2992T allele, either in the mother or offspring, confers reduced fetal growth restraint particularly among first-born infants as indicated by larger birth size and less postnatal compensatory catch-up weight gain.

In subsequent pregnancies and in larger babies, a mendelian pattern of inheritance of birthweight is observed [27]. It was in these second and subsequent pregnancies that we saw association between size at birth and common allele class at the insulin gene minisatellite (*INS *VNTR) [28], which also regulates *IGF2 *expression [29]. Offspring *INS *VNTR class III alleles were associated with larger head size and higher IGF-II levels at birth, and this effect was masked in growth-restricted first pregnancies [28]. Thus, there may be important interaction between maternal parity and imprinted and non-imprinted genes involved in the regulation of IGF-II expression and fetal growth.

Altogether we identified ten SNPs in *H19*. Given that there is no confirmed function for the *H19 *transcript *in vivo *[30], it is difficult to assess the effect of the *H19 *2992 SNP. It is possible that this variant: (a) may alter the RNA itself, and might influence the putative growth suppressing function of the *H19 *RNA; (b) may influence levels of *H19 *transcripts, leading to an effect as above; (c) may be in linkage with an unidentified regulatory element in *H19 *acting on *IGF2 *in cis; (d) may be in linkage with variants in known cis regulatory elements for *IGF2*, such as the insulator or the enhancers. Further work, including systematic genotyping of other SNPs in the extended region and functional studies, will be required to identify the causal variant(s) and, hence, possible mechanisms by which they may influence birthweight and cord blood IGF-II levels. The identification of separate effects of *H19 *on fetal growth, by acting both within the mother and also within the fetus, could be confirmed by studying a larger population to provide greater power to detect an interaction between mother's and offspring's genotypes, and by exploration of grand-parental genotype and allele transmission to the mother. Intriguingly, while we confirmed that the offspring 2992 genotype (CC vs. CT) association with birthweight was independent of mother's genotype (within CC mothers; Table 4), that finding also raises the surprising suggestion of a paternal *H19 *allele transmission effect, however, we did not detect any formal association with paternal transmission. An alternative explanation would be that the *H19 *2992 is in LD with another paternally transmitted determinant of birthweight. Further studies with larger numbers of complete informative trios will be needed to clarify this issue.

## Conclusion

In conclusion, the common *H19 *2992T allele, in the mother or offspring or both, may confer reduced fetal growth restraint, as indicated by associations with larger offspring birth size, higher cord blood IGF-II levels, and lower compensatory early postnatal catch-up weight gain. These observations may have implications for the early origins of adult disease hypothesis [31]. Following the original observations of association between small size at birth and increased later risks for cardiovascular disease and diabetes [32], recent studies have highlighted that these risks are strongest in subjects with the combination of fetal growth restraint followed by rapid postnatal catch-up growth [33]. Common factors that lead to fetal growth restraint in humans include mother's smoking and 1^st ^pregnancies [5]. Common variants in exclusively maternally-expressed genes (such as *H19*) or maternally transmitted genes (such as in mitochondrial DNA) [7] could also contribute to fetal growth restraint and compensatory postnatal catch-up growth.

## Methods

### Subjects

The Avon Longitudinal Study of Parents and Children (ALSPAC) birth cohort numbers 14,185 children comprising over 80% of all births in three Bristol based health authorities between April 1991 and December 1992 [34]. Our studies are confined to a 10% sub-cohort ("Children in Focus") [4] and a second (control) cohort for which there were detailed data on size at birth and subsequent childhood growth. In these families, offspring DNA was extracted from cord blood samples collected at birth, mothers DNA from venous blood samples in pregnancy, and fathers DNA from mouthswab samples. *H19 *genotypes were analysed in available DNA samples from 1,696 children, 822 mothers and 661 fathers. Cord blood samples were collected at birth in 338 of these infants and assayed for IGF-II levels as previously reported [26]; these infants had larger birthweight (mean = 3546 g) than infants for whom cord blood was unavailable (3467 g), but were no different in H19 +2992 genotype (chi-square: P = 0.7)

The Cambridge (Wellbeing) birth cohort was studied to provide independent confirmation of mother's *H19 *genotype associations. This cohort was recruited from full-term, singleton deliveries at the Rosie Maternity Unit, Cambridge, 1999 to 2000. Routinely collected clinical data were available on offspring birthweight and mother's whole blood glucose levels measured at one hour following a standard dose oral glucose load at 27 to 32 weeks of gestation. Mother's were selected of full-term, singleton infants. Exclusion criteria were mother's hypertension and treatment for diabetes during pregnancy. We sought permission from the mother's General Practitioner to approach the mother to collect her DNA sample by postal mouthswab kits and questionnaire data on her other children. Mother's DNA was extracted and genotyped for the *H19 *2992 genotype only. Local ethical committee approval and signed informed consent from mothers were obtained.

### SNP identification

In an independent cohort of 20 subjects, ten SNPs were identified in the *H19 *region using denaturing high performance liquid chromatography (Wave™) and were characterised by sequencing of PCR products (SNPs submitted to dbSNP). Confirmation and assessment of allele frequency was obtained by genotyping a second independent cohort of 100–200 subjects. We selected three *H19 *SNPs as these SNPs marked different haplotypes (2992 C/T [rs217727], 1737 A/G [rs2067051], and 3238 A/G [rs2839703]; the SNP numbers relate to their position in accession number M32053). We genotyped these three SNPs in offspring DNA samples from the ALSPAC cohort for genetic association studies. The *H19 *2992 SNP was subsequently genotyped in DNA samples from ALSPAC parents, and from mothers in the Cambridge cohort.

### *H19 *2992 C/T genotyping

Genomic DNA (40 ng) was amplified in a reaction mixture containing 1x ammonium reaction buffer, 50 μM each dNTP, 500 μM magnesium, 225 ng each primer (forward: 5'-aaagacaccatcggaacagc-3', reverse: 5'-agcttccagact aggcgagg-3'), 10% (v/v) dimethylsulphoxide and 0.6 units DNA polymerase (Bioline, London, UK) in a 45 μL reaction volume. After an initial 5 min. incubation at 94°C, twenty amplification cycles of 94°C (65 sec.), 58°C (65 sec., dropping 0.5°C per cycle) and 72°C (140 sec.) were performed. This was followed by 14 cycles of 94°C (65 sec.), 48°C (65 sec.) and 72°C (140 sec.) and a final incubation of 72°C for 10 min. After overnight digestion with *Fnu*4HI (2 units per reaction; New England Biolabs, Hitchin, UK) at 37°C, PCR products were separated by agarose gel electrophoresis. This gave a 351 bp band for the 'T' allele and a 304 bp band for the 'C' allele. The observed genotype frequencies: CC: 65.3%, CT: 31.5%, TT: 3.3% (Table 1) were in Hardy Weinberg equilibrium (chi-square test: P = 0.99). The *H19 *2992 SNP was also genotyped in the parents DNA samples.

### *H19 *1737 A/G genotyping

The reaction mix for the *H19 *1737 genotyping was the same as for the 2992 SNP, with the exception of the oligonucleotide primer sequences (forward: 5'-aaggtgacatcttctcgggg-3'; reverse: 5'-tgagagctcattcactccgc-3'). The amplification conditions were the same as those for the 2992 SNP except that the annealing temperatures were 4°C higher throughout. Overnight digestion with *Tsp*45I (2 units per reaction; New England Biolabs) produced 474 and 131 bp bands for the 'A' allele and 329, 145 and 131 bp fragments for the 'G' allele. The observed genotype frequencies (AA: 23.1%, AG: 52.6%, GG: 24.4%) were in Hardy Weinberg equilibrium (chi-square test: P = 0.5).

### *H19 *3238 A/G genotyping

The reaction mix for the *H19 *3238 genotyping was the same as for the 2992 SNP, with the exception of the oligonucleotide primer sequences (forward: 5'-aaagacaccatcggaacagc-3'; reverse: 5'-agcttccagactaggcgagg-3'). Amplification conditions were the same as those for the 2992 SNP except that the annealing temperatures were 4°C higher throughout. Overnight digestion with *Dde*I (2 units per reaction; New England Biolabs) produced 137 and 44 bp bands and multiple small bands (116, 76, 60 and 26 bp) for the 'A' allele and a 181 bp band along with the same multiple small bands for the 'G' allele. The observed genotype frequencies (AA: 38.0%, AG: 48.5%, GG: 13.5%) were in Hardy Weinberg equilibrium (chi-square test: P = 0.8).

### Microsatellite validation of parental DNA samples

Parental DNA samples were validated using amplification of markers D16S539, D7S820, D13S317 and D5S818 (Geneprint Multiplex-GammaSTR kit, Promega, Southampton, UK) on an ABI 3700 instrument as previously described [28]. We had DNA samples from 597 complete and validated parent-offspring trios.

### Calculations and statistics

Postnatal weight and length gains were calculated by transforming weight and length data into SD scores allowing for variation relating to (gestational) age and gender, using the formula [SD score = individual value – group mean / group SD] [35], and then calculating changes in weight and length SD score between birth to 3 years.

Associations between offspring or mother's genotype and size at birth were determined by ANOVA. Multivariate analysis (general linear models) was used to identify independent effects of mother's and offspring genotypes. Associations with *H19 *2992 allele transmission were assessed by TDT using both quantitative and qualitative methods. Parent-of-origin effects were sought using quantitative and qualitative methods, allowing for possible confounding by the effects of non-transmission of mother's alleles [36,37].

## Abbreviations

**ALSPAC **– Avon Longitudinal Study of Parents and Children

**IGF **– insulin-like growth factor

**Primip **– primiparous

**SNP **– single nucleotide polymorphism

## Authors' contributions

CP performed genotyping and drafted the manuscript. KO contributed to the study design, and co-ordination, performed statistical analyses and drafted the manuscript. BB performed SNP identification, and contributed to the study design. DW performed Cambridge cohort recruitment, DNA preparation and genotyping. HC performed statistical analyses. SR supervised preparation of ALSPAC DNA samples and validation of genotyping results. MR participated in the design and co-ordination of the ALSPAC study, and supervised DNA management. WR participated in the conception and design of the study. JT supervised SNP identification, genotyping and data analyses. DD was responsible for the study conception, and contributed to the study coordination, analyses and drafting the manuscript. All authors read and approved the final manuscript.

## Supplementary Material

Additional File 1 Birthweight SD score (adjusted for sex and gestational age) by combination of *H19 *2992/1737/3238 genotypes (for each SNP, 1 = more common homozygote, 2 = heterozygote, 3 = less common homozygote). Click here for file

Additional File 2 Schematic map of *H19 *indicating exons, SNPs published on dbSNP and also published SNPs identified by the Juvenile Diabetes Research Foundation/Wellcome Trust Diabetes and Inflammation Laboratory (DIL) T1Dbase . *H19 *2992 is indicated by the labels rs217727 and DIL1049. Click here for file

## References

[B1] Ounsted M, Ounsted C (1966). Maternal regulation of intrauterine growth. Nature.

[B2] Walton A, Hammond J (1938). The maternal effects on growth and conformation in Shire horse-Shetland pony crosses. Proceedings of the Royal Society of London.

[B3] Joubert DM, Hammond J (1958). A cross breeding experiment with cattle, with special reference to the maternal effect in South Devon-Dexter crosses.. Journal of Agricultural Science.

[B4] Ong KK, Ahmed ML, Emmett PM, Preece MA, Dunger DB, the-ALSPAC-Study-Team (2000). Association between postnatal catch-up growth and obesity in childhood: prospective cohort study. BMJ.

[B5] Ong KK, Preece MA, Emmett PM, Ahmed ML, Dunger DB (2002). Size at birth and early childhood growth in relation to maternal smoking, parity and infant breast-feeding: longitudinal birth cohort study and analysis. Pediatric Research.

[B6] Ounsted M, Scott A, Ounsted C (1986). Transmission through the female line of a mechanism constraining human fetal growth. Annals of Human Biology.

[B7] Casteels K, Ong KK, Phillips DI, Bednarz A, Bendall H, Woods KA, Sherriff A, Golding J, Pembrey ME, Poulton J, Dunger DB, ALSPAC_Study_Team (1999). Mitochondrial 16189 variant, thinness at birth and type 2 diabetes. Lancet.

[B8] Reik W, Walter J (2001). Genomic imprinting: parental influence on the genome. Nature Reviews Genetics.

[B9] Giannoukakis N, Deal C, Paquette J, Goodyer CG, Polychronakos C (1993). Parental genomic imprinting of the human IGF2 gene. Nature Genetics.

[B10] Ohlsson R, Nystrom A, Pfeifer-Ohlsson S, Tohonen V, Hedborg F, Schofield P, Flam F, Ekstrom TJ (1993). IGF2 is parentally imprinted during human embryogenesis and in the Beckwith-Wiedemann syndrome. Nature Genetics.

[B11] Constancia M, Dean W, Lopes S, Moore T, Kelsey G, Reik W (2000). Deletion of a silencer element in Igf2 results in loss of imprinting independent of H19. Nature Genetics.

[B12] Murrell A, Heeson S, Bowden L, Constancia M, Dean W, Kelsey G, Reik W (2001). An intragenic methylated region in the imprinted Igf2 gene augments transcription. EMBO Reports.

[B13] Leighton PA, Saam JR, Ingram RS, Stewart CL, Tilghman SM (1995). An enhancer deletion affects both H19 and Igf2 expression. Genes & Development.

[B14] Davies K, Bowden L, Smith P, Dean W, Hill D, Furuumi H, Sasaki H, Cattanach B, Reik W (2002). Disruption of mesodermal enhancers for Igf2 in the minute mutant. Development.

[B15] Murrell A, Heeson S, Reik W (2004). Interaction between differentially methylated regions partitions the imprinted genes Igf2 and H19 into parent-specific chromatin loops. Nature Genetics.

[B16] Thorvaldsen JL, Duran KL, Bartolomei MS (1998). Deletion of the H19 differentially methylated domain results in loss of imprinted expression of H19 and Igf2. Genes & Development.

[B17] Hark AT, Schoenherr CJ, Katz DJ, Ingram RS, Levorse JM, Tilghman SM (2000). CTCF mediates methylation-sensitive enhancer-blocking activity at the H19/Igf2 locus. Nature.

[B18] Bell AC, Felsenfeld G (2000). Methylation of a CTCF-dependent boundary controls imprinted expression of the Igf2 gene. Nature.

[B19] Hao Y, Crenshaw T, Moulton T, Newcomb E, Tycko B (1993). Tumour-suppressor activity of H19 RNA. Nature.

[B20] Li YM, Franklin G, Cui HM, Svensson K, He XB, Adam G, Ohlsson R, Pfeifer S (1998). The H19 transcript is associated with polysomes and may regulate IGF2 expression in trans. Journal of Biological Chemistry.

[B21] Leighton PA, Ingram RS, Eggenschwiler J, Efstratiadis A, Tilghman SM (1995). Disruption of imprinting caused by deletion of the H19 gene region in mice. Nature.

[B22] Stylianopoulou F, Efstratiadis A, Herbert J, Pintar J (1988). Pattern of the insulin-like growth factor II gene expression during rat embryogenesis. Development.

[B23] Wu HK, Squire JA, Song Q, Weksberg R (1997). Promoter-dependent tissue-specific expressive nature of imprinting gene, insulin-like growth factor II, in human tissues. Biochemical and Biophysical Research Communications.

[B24] Zapf J (1994). Role of insulin-like growth factor II and IGF binding proteins in extrapancreatic tumor hypoglycemia. Hormone Research.

[B25] Catalano PM, Thomas AJ, Huston LP, Fung CM (1998). Effect of maternal metabolism on fetal growth and body composition. Diabetes Care.

[B26] Ong KK, Kratzsch J, Kiess W, Costello M, Scott CD, Dunger DB, the-ALSPAC-Study-Team (2000). Size at birth and cord blood levels of insulin, insulin-like growth factor I (IGF-I), IGF-II, IGF-binding protein-1 (IGFBP-1), IGFBP-3, and the soluble IGF-II/mannose-6-phosphate receptor in term human infants.. Journal of Clinical Endocrinology & Metabolism.

[B27] Ounsted M, Scott A, Moar VA (1988). Constrained and unconstrained fetal growth: associations with some biological and pathological factors. Annals of Human Biology.

[B28] Ong KK, Petry CJ, Barratt BJ, Ring S, Cordell HJ, Wingate DL, Pembrey ME, Todd JA, Dunger DB (2004). Maternal-fetal interactions and birth order influence insulin variable number of tandem repeats allele class associations with head size at birth and childhood weight gain. Diabetes.

[B29] Paquette J, Giannoukakis N, Polychronakos C, Vafiadis P, Deal C (1998). The INS 5' variable number of tandem repeats is associated with IGF2 expression in humans. Journal of Biological Chemistry.

[B30] Jones BK, Levorse JM, Tilghman SM (1998). Igf2 imprinting does not require its own DNA methylation or H19 RNA. Genes & Development.

[B31] Barker DJ (1992). Fetal growth and adult disease. B J Obstet Gynaecol.

[B32] Hales CN, Barker DJ, Clark PM, Cox LJ, Fall C, Osmond C, Winter PD (1991). Fetal and infant growth and impaired glucose tolerance at age 64. BMJ.

[B33] Eriksson JG, Forsen T, Tuomilehto J, Winter PD, Osmond C, Barker DJ (1999). Catch-up growth in childhood and death from coronary heart disease: longitudinal study. BMJ.

[B34] Golding J, Pembrey ME, Jones R (2001). ALSPAC--the Avon Longitudinal Study of Parents and Children. I. Study methodology. Paediatric and Perinatal Epidemiology.

[B35] Cole TJ (1994). Do growth chart centiles need a face lift?. BMJ.

[B36] Whittaker JC, Gharani N, Hindmarsh P, McCarthy MI (2003). Estimation and testing of parent-of-origin effects for quantitative traits. American Journal of Human Genetics.

[B37] Gauderman WJ (2003). Candidate gene association analysis for a quantitative trait, using parent-offspring trios. Genetic Epidemiology.

