# Supplementary figures and images for: Common polymorphism in H19 associated with birthweight and cord blood IGF-II levels in humans
Source: BMC Genet. 2005 May 10;6:22. doi: 10.1186/1471-2156-6-22 (PMC1140752; doi:10.1186/1471-2156-6-22)

## Slide 1
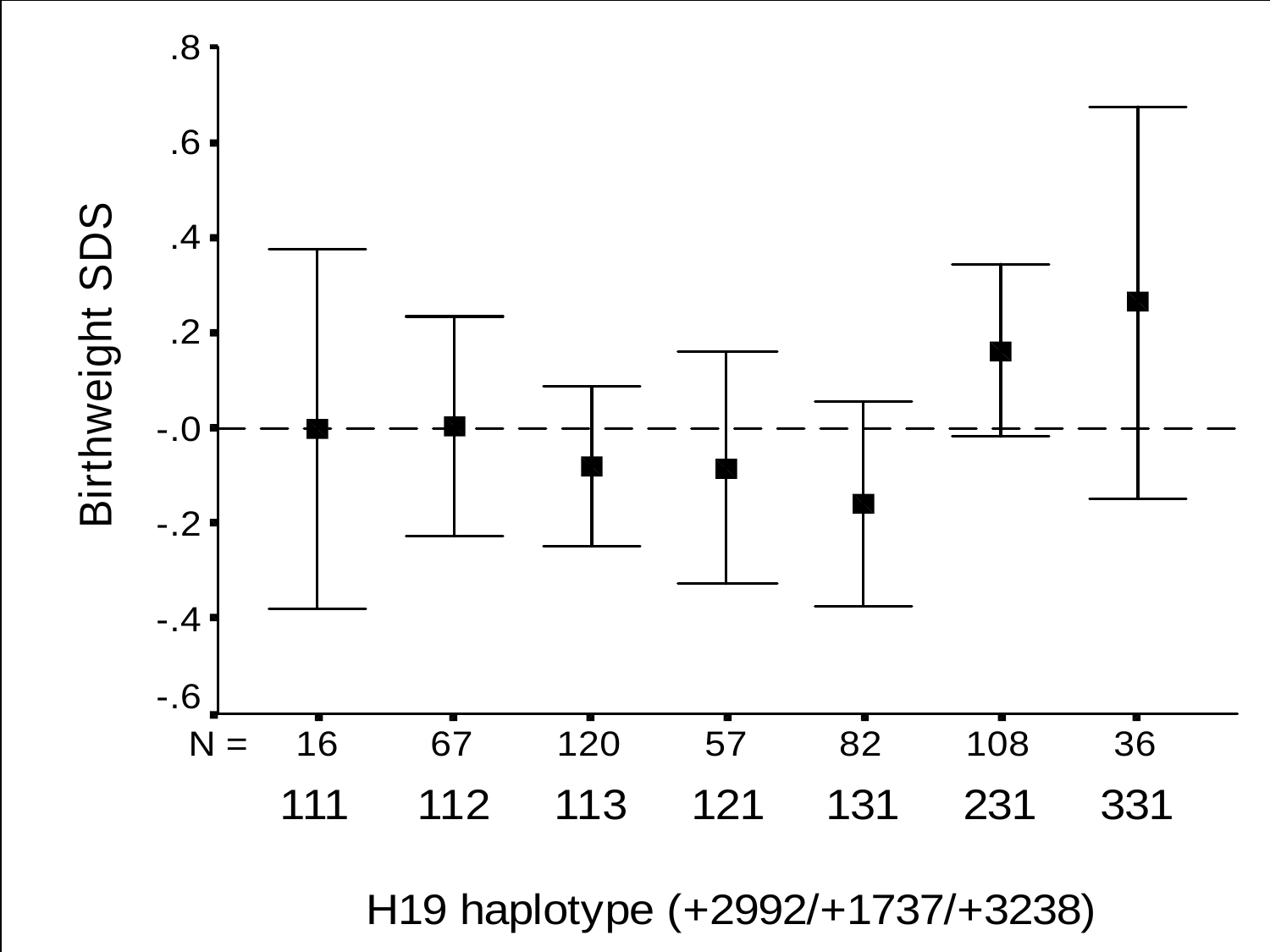

Supplement: Additional File 1 — Birthweight SD score (adjusted for sex and gestational age) by combination of H19 2992/1737/3238 genotypes (for each SNP, 1 = more common homozygote, 2 = heterozygote, 3 = less common homozygote). [file 1471-2156-6-22-S1.ppt]

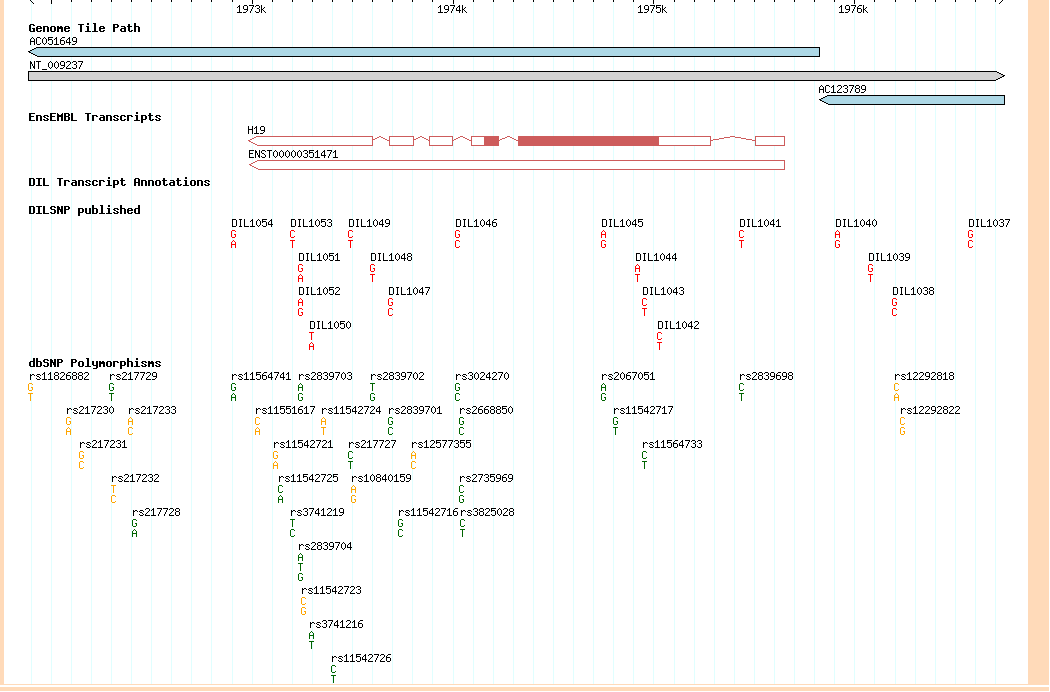

Supplement: Additional File 2 — Schematic map of H19 indicating exons, SNPs published on dbSNP and also published SNPs identified by the Juvenile Diabetes Research Foundation/Wellcome Trust Diabetes and Inflammation Laboratory (DIL) T1Dbase . H19 2992 is indicated by the labels rs217727 and DIL1049. [file 1471-2156-6-22-S2.bmp]
